# C-955-01. Advanced Practice Provider-Administered Moderate Sedation: A Novel Approach to Burn Wound Care

**DOI:** 10.1093/jbcr/irag033.150

**Published:** 2026-04-06

**Authors:** Amber Kohler, Jennifer Rodgers, Scott W Mueller, Arek J Wiktor

**Affiliations:** Burn & Frostbite Center at UCHealth, University of Colorado Hospital, Aurora, CO; Burn & Frostbite Center at UCHealth, University of Colorado Hospital, Aurora, CO; Burn & Frostbite Center at UCHealth, University of Colorado Hospital, Aurora, CO; Burn & Frostbite Center at UCHealth, University of Colorado Hospital, Aurora, CO

## Abstract

**Introduction:**

Moderate sedation is commonly utilized to alleviate the significant pain and anxiety associated with burn wound care. Variability in practice patterns can lead to safety concerns and inconsistent comfort among providers. To address these issues, a new practice protocol was developed enabling advanced practice providers (APPs) to administer moderate sedation for burn wound care, ensuring regulatory compliance and standardization in the delivery model. We aimed to assess the feasibility of this practice.

**Methods:**

A 12-month retrospective review was conducted at our ABA-verified burn center, evaluating the first year of APP-administered moderate sedation. APPs underwent comprehensive training in collaboration with the anesthesia assistant program, covering pharmacokinetics, end tidal CO2 monitoring, and airway management. Education on medication administration and documentation was provided, and computer training modules were completed for credentialing in moderate sedation. APPs were monitored in two successful sedations before practicing independently. Patient charts were reviewed for demographics, medications administered prior to and during sedation, duration and depth of sedation, and adverse events.

**Results:**

APPs performed 105 sedations on 26 burn and soft tissue injury patients with natural airways; 70% were male (median age, 42 years; median %TBSA, 12; median ASA score, 2). Patients received a median of three oral premedications (morphine equivalents 20 mg [15, 22.5], lorazepam 1 mg [1, 1], oral ketamine 100 mg [100, 150], and/or sublingual dexmedetomidine 195mcg [130, 200]) and three IV push medications (fentanyl 150mcg [100, 200], midazolam 1 mg [0.5, 1.5], ketamine 50 mg [30, 72], and/or dexmedetomidine infusion 0.8 mcg/kg/hr [0.8, 1]). A median of three sedations per patient lasted a median of 52 minutes (range 25-165). The median Richmond Agitation Sedation Scale (RASS) achieved was -2. One patient reached deep sedation (RASS -4), managed by decreasing the dexmedetomidine infusion; no airway or cardiovascular compromise occurred, and the patient recovered spontaneously. No reversal agents (naloxone or flumazenil) were required. Hypertension was recorded in 17 sedations (one requiring treatment), and hypotension in six (all managed by reducing the dexmedetomidine infusion). No intubations, cardiopulmonary resuscitation, or other adverse events were reported, including those related to hypoxia or aspiration.

**Conclusions:**

Specially trained burn APPs can administer moderate sedation for burn wound care, expanding their scope of practice and providing a feasible model for standardized sedation delivery.

**Applicability of Research to Practice:**

Burn centers should consider implementing APP training programs to support the delivery of moderate sedation within their full scope of practice.

**Funding for the Study:**

N/A.

